# Total and Extracellular Vesicle cAMP Contents in Urine Are Associated with Autosomal Dominant Polycystic Kidney Disease (ADPKD) Progression

**DOI:** 10.3390/life13091817

**Published:** 2023-08-28

**Authors:** María Lucía Rosenberg, Agustín Yaneff, Gonzalo Manuel Ferradás, Margarita Paz Villafañe Tapia, Carlos Alberto Davio, Nora Paula Goette, Sandra Gabriela Vlachovsky, Roxana Noemí Peroni, Elisabet Mónica Oddo, Pablo Javier Azurmendi

**Affiliations:** 1 Instituto de Investigaciones Médicas Alfredo Lanari, Facultad de Medicina, Universidad de Buenos Aires (UBA), Buenos Aires 1427, Argentinagoette.nora@lanari.uba.ar (N.P.G.);; 2 Instituto de Investigaciones Médicas, UBA—Consejo Nacional de Investigaciones Científicas y Técnicas (IDIM, UBA-CONICET), Facultad de Medicina, Universidad de Buenos Aires, Buenos Aires 1427, Argentina; 3 Instituto de Investigaciones Farmacológicas (ININFA-UBA-CONICET), Facultad de Farmacia y Bioquímica, Universidad de Buenos Aires, Buenos Aires 1113, Argentinarperoni@ffyb.uba.ar (R.N.P.); 4 Departamento de Farmacología, Facultad de Farmacia y Bioquímica, Universidad de Buenos Aires, Buenos Aires 1113, Argentina

**Keywords:** urine extracellular vesicles, cystic growth, ADPKD progression, cyclic AMP

## Abstract

ADPKD is the most common genetic renal disease, characterized by the presence of multiple cysts which, through slow and gradual growth, lead to glomerular filtration rate (GFR) decline and end-stage renal disease. Cystic growth is associated with increased intracellular levels of 3′,5′-cyclic adenosine monophosphate (cAMP). Extracellular vesicles (EVs) are proposed to participate in “remote sensing” by transporting different cargoes, but their relevance to ADPKD progression is poorly understood. This study aimed to determine whether cAMP is contained in urinary EVs and, if so, how total and/or EV cAMP contents participate in disease progression. Fourteen ADPKD patients, naïve for V_2_ receptor antagonism treatment, and seven controls were studied. Progression was evaluated by estimating GFR (eGFR) and height-adjusted total kidney volume (htTKV). Fresh morning urine was collected to determine cAMP by the competitive radioligand assay. Urine EVs were isolated using an adapted centrifugation method and characterized by electron microscopy, dynamic light scanning, flow cytometry with FITC CD63 labeling, protein and RNA content, and *AQP2* and *GAPDH* mRNA detection. Total and EV cAMP was measurable in both control and patient urine samples. Total cAMP was significantly correlated with eGFR and its annual change but inversely correlated with htTKV. The cAMP-EVs showed a bimodal pattern with htTKV, increasing to ~1 L/m and falling at larger sizes. Our results demonstrate that urine cAMP correlates with ADPKD progression markers, and that its extracellular delivery by EVs could reflect the architectural disturbances of the organ.

## 1. Introduction

Polycystic kidney disease (PKD) is characterized by fluid-filled cysts that compress the surrounding renal parenchyma and compromise renal function. Autosomal dominant PKD (ADPKD) is one of the most common genetic diseases, affecting 1 in 1000 adults worldwide and being the first genetic cause of kidney disease and the fourth of end-stage renal disease (ESRD). This pathology is characterized by multiple epithelial cysts that, through slow and gradual growth, lead to a large expansion of the total kidney volume (TKV). Fluid accumulation within the cysts and the proliferation of cystic cells are the two main mechanisms for this expansion. The primary sources of the fluid are both non-reabsorbed glomerular filtrate and transepithelial secretion across the monolayer cystic cell membrane, which result in net fluid and solute secretion into the cyst. The proliferation rate of cystic cells is only slightly higher than that of normal tubular cells but is sufficient to increase the organ size by ~5% per year [1]. After a long phase of normal renal function and arterial hypertension, the glomerular filtration rate (GFR) falls relatively abruptly and steadily, leading to ESRD [2]. Considerable variability in disease severity among affected individuals includes intrafamilial variability in renal progression, which cannot be explained by already-known factors [3].

Most studies in ADPKD animal models, cell lines, and patient tissue have revealed that abnormal polycystins due to mutations in *PKD1* or *PKD2* genes reduced basal intracellular calcium levels. *PKD1* encodes polycystin-1, a transmembrane mechano-sensor receptor, whereas *PKD2* encodes polycystin-2, a non-selective cationic channel permeable to calcium. In the primary cilium, the polycystin-1 and -2 complex regulates cell calcium influx. Then, the complex disruption due to mutated proteins disturbs the calcium cellular homeostasis [4]. This reduction in cytosolic calcium level is associated with increased levels of 3′,5′-cyclic adenosine monophosphate (cAMP) in cystic tissues [5]. cAMP is a second messenger synthesized by adenylyl cyclases in response to the activation of membrane G protein-coupled receptors, and its intracellular concentration modulates the protein kinase A (PKA) pathway. Several pieces of evidence have demonstrated that cAMP-dependent PKA activation promotes both cell proliferation and fluid secretion into the cyst sac. PKA stimulates cell proliferation by the B-Raf/MEK/ERK signaling pathway, whereas it promotes cyst fluid accumulation through chloride-dependent secretion by the cystic fibrosis transmembrane conductance regulator (CFTR) [6,7]. Then, cAMP and PKA signaling enhances several proliferative pathways in cells derived from polycystic kidneys while inhibiting proliferation in cells derived from the normal human renal cortex [6], and precise regulation of intracellular cAMP levels is critical. Since cAMP overproduction within cells leads to exacerbated stimulation of certain secretory events, dysregulation of cell function, or even cell toxicity [8], several hormones such as parathormone, somatostatin, and vasopressin regulate nephrogenic production under physiological conditions [9]. In this sense, the blockage of vasopressin action through the V_2_ receptor ameliorated the cyst enlargement and kidney growth via downregulation of adenylyl cyclase-dependent cAMP production in a polycystic disease murine model [10]. Clinical trials confirmed the aforementioned beneficial effect of the V_2_ receptor antagonist tolvaptan in ADPKD patients, becoming the only currently available treatment to ameliorate cyst growth and, hence, renal function decline in patients with rapid disease progression [11]. Although the pivotal role in the pathophysiology and clinical progression of ADPKD is well documented, precise tools to monitor cAMP through disease evolution and treatment effectiveness need further investigation.

It is well known that cAMP also plays a key role in the extracellular regulation of fluid homeostasis in epithelial cells from the intestine, kidney, and lung [12], but the cell-to-cell delivery of cAMP from renal tubules, as well as from cystic epithelia, is poorly understood. In this sense, extracellular vesicles (EVs) are released from the plasma membrane of almost every mammalian cell, and growing evidence shows that EVs participate in the maintenance of homeostasis and in different pathological processes. The term EVs mainly includes exosomes, microvesicles, and apoptotic bodies. Exosomes are vesicles (50–100 nm in diameter) generated by the exocytosis of multivesicular bodies (MVBs). They participate in the direct contact between surface molecules of recipient cells, endocytosis of vesicles, and vesicle–cell membrane fusion. The main functions of exosomes are antigen presentation and immunostimulatory and inhibitory activities through horizontal transfers of their cargoes. Microvesicles are larger extracellular membrane vesicles (100–1000 nm in diameter) that participate in procoagulant activity and fetomaternal communication and contribute to the oncogenic cellular transformation and pro-invasive character of tumors. Apoptotic bodies (1–5 μm in diameter) are EVs released as blebs of cells undergoing apoptosis that participate in antigen presentation to immune cells, especially in immunosuppression processes [13]. EVs are proposed to participate in “remote sensing”. This is cell-to-cell communication by transporting different cargoes involving several components of the cell of origin that vary in pathophysiological conditions [14]. Although cystic cell proliferation has been related to elevated cAMP [6], it is not known whether cell-to-cell delivery of this second messenger as EV cargo has a role in cyst expansion and kidney function decline.

This study aimed to determine whether cAMP is contained in urinary EVs from ADPKD patients and healthy controls, and whether total and/or EV cAMP contents are related to disease progression.

## 2. Materials and Methods

### 2.1. Study Design

Both ADPKD patients and control subjects were recruited at the Instituto de Investigaciones Médicas Alfredo Lanari of Universidad de Buenos Aires, Buenos Aires, Argentina. This study is part of an observational, single-center, and longitudinal protocol [15,16] that was approved by the institutional Ethics Committee, and each subject gave written informed consent. Adult patients with a positive diagnosis independent of blood pressure (BP) status and absence of overt proteinuria, who attended the regular annual visit from April to December 2022, were enrolled in this study. ADPKD was diagnosed by ultrasound (at least two unilateral or bilateral cysts in individuals under 30 years of age, two cysts in each kidney in individuals 30–59 years of age, and four cysts in each kidney in individuals 60 years of age or older) according to established criteria [17], clinical features (i.e., flank pain, hypertension, hematuria, proteinuria, or kidney function impairment), and family history of ADPKD [9]. In order to exclude other possible modifier causes not related to natural ADPKD evolution of urine cAMP levels, any other concomitant renal disease and V_2_ receptor antagonism treatment were established as exclusion criteria.

Subjects with no ADPKD were also recruited as a control group for cAMP and EV studies. The clinical and biochemical parameters evaluated for each participant were as follows: anthropometric, blood pressure and medication registries, estimated GFR (eGFR) by plasma creatinine, TKV, total protein, albumin, and creatinine in urine. We measured the total and EV cAMP contents of both groups from fresh midway urine. In addition, the ADPKD clinical parameters were compared to total and EV cAMP levels to find their association with disease progression.

### 2.2. BP Measurements

Office BP registeries were obtained using an aneroid sphygmomanometer (Durashock DS44 Adult, Welch Allyn^®^, Skaneateles Falls, NY, USA) before eating breakfast, smoking, or consuming caffeine, as previously described [15]. The average of the second and third seated measurements was used for further analysis.

### 2.3. Total Kidney Volume (TKV)

TKV was calculated using the ellipsoid equation [16] by taking the length, width, and thickness dimensions of each kidney image acquired with an Aquilion 16 computer tomography scanner (Toshiba, Tokio, Japan), and it was expressed as height-adjusted TKV (htTKV) for further comparisons. Patients were classified into 1A–1E subclasses considering htTKV according to the imaging classification of ADPKD from the Mayo Clinic developed by the Mayo Foundation and Medical Education and Research [11].

### 2.4. Urine and Blood Sample Collection

From the recruited participants, fresh morning urine samples were collected, and blood was extracted with sodium heparin as an anticoagulant. The samples were stored at 4 °C for immediate use or at −80 °C for longer periods. In addition, an aliquot of the same urine sample was separated to perform EV isolation. Midstream urine with abnormal microscopy results in sediment was discarded, as previously described [15].

### 2.5. Urine EV Isolation

The gold standard for EV isolation is 200,000× *g* ultracentrifugation for 1 h at 4 °C, a method currently not available to clinical laboratories. Therefore, we isolated EVs from morning urine using an adapted-centrifugation method, equalizing time and speed (50,000× *g* for 4 h at 4 °C) to the ultracentrifugation method and following the current criteria for collection, storage processing, and normalization written by the Urine Task Force of the Rigor and Standardization Subcommittee of International Society of Extracellular Vesicles (ISEV) [18].

**Figure 1:**
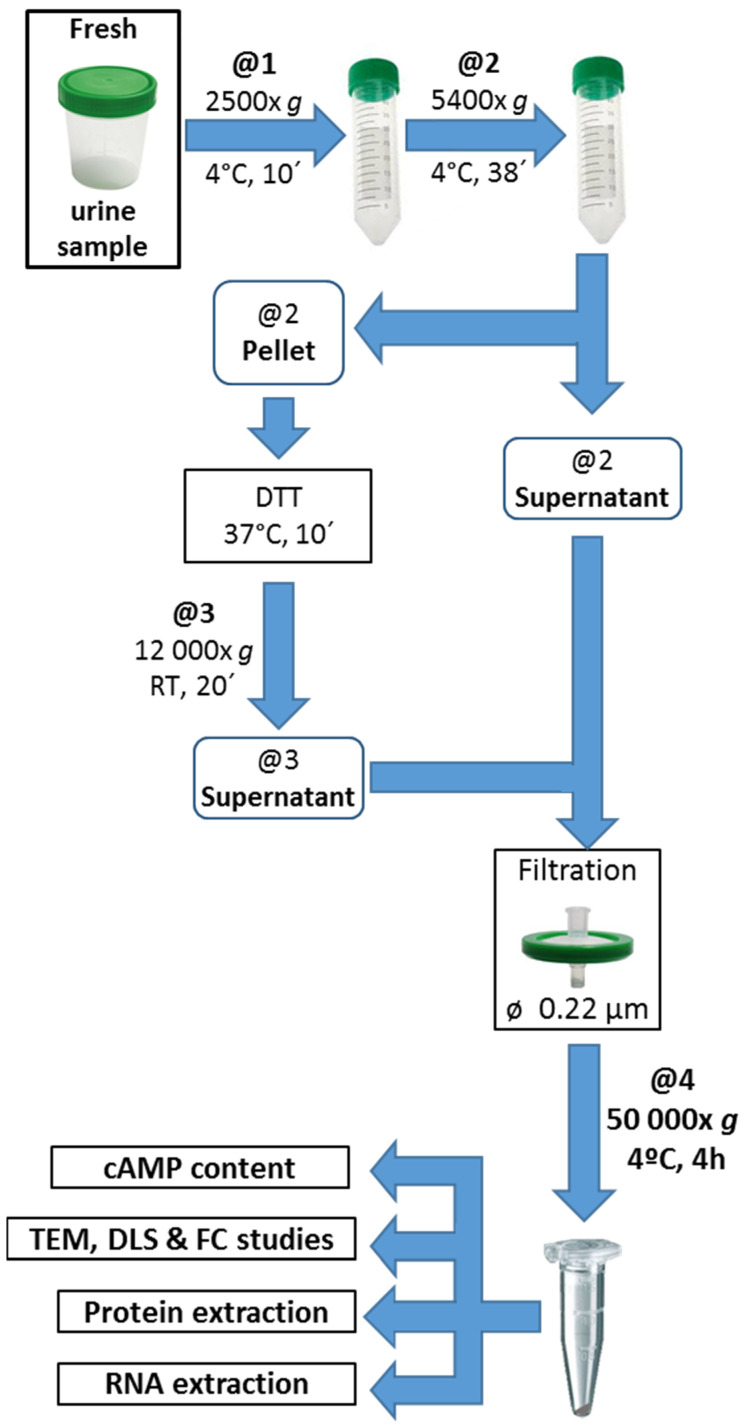
Isolation of urinary extracellular vesicles (EVs) using the adapted centrifugation method. First-morning-void urine samples were collected in sterile recipients. A 50 mL aliquot was pretreated by centrifugation (@1) to pull down cell fragments and apoptotic cells, the pellet was discarded, and the supernatant was centrifuged again (@2). The resulting pellet was incubated with 200 mg/mL dithiothreitol (DTT) and centrifuged (@3); then, the supernatants from @2 and @3 were combined. After a cleaning step with a 0.22 µm syringe filter, the pretreated urine was then centrifuged by equalizing time and speed (@4) to the traditional ultracentrifugation method. Finally, the pellet was reconstituted with PBS for cAMP and morphometric determination [transmission electron microscopy (TEM), dynamic light scanning (DLS)], and flow cytometry (FC) analyses, or lysis buffers for protein and RNA extraction. RT: room temperature.

Urine aliquots of 50 mL collected in sterile recipients were pre-processed with two centrifugation steps, the first one being at 2500× *g* for 10 min at 4 °C to pull down cell fragments and apoptotic cells. The supernatant was centrifuged at 5400× *g* for 38 min at 4 °C with a Thermo Scientific^®^ Sorvall^®^ ST 16 (Thermo Electron LED GmbH, Langenselbold, Germany) centrifuge. The resulting supernatant was stored at 4 °C, and the pellet was treated with 200 mg/mL of dithiothreitol (DTT; Promega Inc., Fitchburg, WI, USA) at 37 °C for 10 min and vortexed every 2 min to disaggregate uromodulin-trapped (e.g., Tamm Horsfall protein) EVs [19]. The DTT-treated pellets were centrifuged at 12,000× *g* for 20 min at room temperature, and the resulting supernatant was combined with the second centrifugation supernatant. To improve the cleaning, the removal of large EVs was performed by filtration with 0.22 µm diameter polyethersulfone membrane (Millex-GP, Merck Millipore Ltd., Carrigtwohill, Ireland). Then, pretreated urine was centrifuged at 50,000× *g* for 4 h at 4 °C with a high-speed refrigerated benchtop centrifuge (Neofuge 23R, Heal Force, Shanghai Lishen Scientific Equipment Co., Ltd., Shanghai, China) (Figure 1). The supernatant was discarded, and the final pellet of the adapted-centrifugation method (FPCM) was reconstituted with appropriate buffers for the subsequent analysis and stored at 4 °C for immediate use or at −80 °C for longer periods, as recommended [20].

### 2.6. EV Characterization

We characterized the vesicles according to size, shape, content, and EV protein-membrane marker. Dynamic light scattering (DLS) and transmission electron microscopy (TEM) methods tested the particles’ complexity and size, respectively. The hydrodynamic average diameter was determined by DLS on 1/20 dilution of fresh FPCM in PBS (final volume of 1 mL) using Zetasizer Nano (Malvern Panalytical Ltd., Malvern, UK) with Non-Invasive Back Scatter (NIBS^®^) technology (Malvern Ltd., Malvern, UK). The determinations were performed at a fixed scattering angle of 173° and fixed laser position of 4.65 mm, using a He–Ne laser (633 nm) and a digital correlator (ZEN3600).

TEM was carried out using a Zeiss EM 109T (Zeiss group, Oberkochen, Germany). Briefly, fresh FPCM was diluted at 10% in PBS (Figure 1) and fixed with 3% glutaraldehyde/PBS; 10 μL of the solution was air-dried in a Petri dish. Then, the grid with a membrane was deposited on the drop and left to rest for 3–5 min, dried with filter paper for 10 min, and incubated for 3–5 min with 2% uranyl acetate. After a wash with bidistilled water and drying, the grid was ready for the TEM scan [21].

EV protein-membrane marker identification was performed by flow cytometry using an FITC-labeled anti-human CD63 mouse antibody (BD Pharmingen, BD Bioscience, San Jose, CA, USA). An aliquot of the FPCM in PBS was incubated in darkness at 37 °C for 15 min with the conjugated antibody or the IgG1 isotype control and fixed with formaldehyde for 20 min. Data acquisition was performed in FACS Canto II flow cytometer (Becton Dickinson Biosciences, San Jose, CA, USA), and the results were analyzed and visualized with Flowing Software version 2.5.1 (Turku Bioscience, Turku, Finland). The histogram data were analyzed and expressed as a percentage of CD63-positive particles subtracting the respective control isotype signal.

The FPCM was dissolved in lysis buffer (50 mM Tris–HCl, 150 mM NaCl, 1 mM EDTA, 1% *v*/*v* NP-40, and 0.25% *w*/*v* sodium-deoxycholate, pH 7.5) containing a protease inhibitor cocktail (Halt Protease Inhibitor Cocktail, Thermo Fisher Scientific, Waltham, MA, USA) (Figure 1), and protein concentration was measured by the Bradford method using the Bio-Rad Protein Assay Dry Reagent (Bio-Rad Laboratories Inc., Hercules, CA, USA) and expressed as μg/μL of urine. For RNA content determination, the FPCM was dissolved in lysis buffer and extracted by Bio-Zol reagent (Productos Bio-Lógicos, Quilmes, Argentina) following the manufacturer’s instructions. Total RNA was measured by spectrophotometric absorbance at 260 nm and expressed as ng/μL of urine. Quantitative real-time PCR was performed to detect *GAPDH* and *AQP2* mRNAs in the total RNAs from FPCM. Total RNA was reverse-transcribed into cDNA using the Luna Script RT Super Mix (New England Bio Labs. Inc., Ipswich, MA, USA) with random primers (GE, Amersham Biosciences, Amersham, UK) following the manufacturer’s instructions before real-time quantitative PCR experiments (RT-qPCR). Primer sequences for *GADPH* and *AQP2* mRNAs were designed as already described [16] taking NM_002046.7 and NM_000486.6 from the NCBI/NLM gene database as reference sequences, respectively. The primer sequences and PCR conditions are available upon request. The PCR products were 137 and 146 pb for *GADPH* and *AQP2* mRNAs, respectively. Their identity was checked according to melting profile at the end of RT-qPCR experiments, and size determination was performed by electrophoresis in 4% agarose gel stained with ethidium bromide. RT-qPCR was carried out with Luna Universal qPCR Master Mix (New England Bio Labs. Inc., Ipswich, MA, USA) and specific primers to mRNAs using the LightCycler^®^ 2.0 equipment (Roche Diagnostic GmbH, Mannheim, Germany), according to published procedures [22].

### 2.7. Creatinine, Total Protein, and Albumin (UAE) Determination

Creatinine was determined by the alkaline picrate colorimetric method in plasma and urine samples. The total urinary protein was determined with a quantitative colorimetric method based on the use of pyrogallol red (Proti U/LCR, Wiener lab, Rosario, Argentina). UAE was measured with a competitive chemiluminescent enzyme immunoassay (Immulite/Immulite 1000 Albumin, DPC, Los Angeles, CA, USA). Creatinine, total protein, and albumin determination were carried out as previously described [15,16].

### 2.8. Estimation of GFR

Glomerular filtration rate was estimated (eGFR) by 4-variable MDRD using plasma creatinine values as described elsewhere [15,16]. The degree of chronic kidney disease for each patient was classified as CKD score according to eGFR and albuminuria [23].

### 2.9. cAMP Determination

The cAMP content was determined by a competitive binding assay to specific protein kinase (PKA) as described previously [24]. Briefly, 1 mL of absolute ethanol was added to a urine or FPCM sample previously dissolved in PBS. The alcohol was then evaporated to concentrate cAMP and resuspended in 50 nM Tris/HCl (pH 7.4) and 0.1% BSA for cAMP determination. cAMP content was determined by competition of [^3^H]cAMP for PKA. The assay allows cAMP to be measured within a standard curve in the range of 0.05–25 pmol/mL within a quantitation limit of 0.1 pmol/mL. The cAMP content was expressed as nmol/g creatinine.

### 2.10. Statistical Procedures

The GraphPad Prism version 8.0.2 software package (GraphPad software, Boston, MA, USA) was used for statistical procedures. The normality was tested by the Shapiro–Wilk test. As most of the quantitative variables were normally distributed, ANOVA or unpaired Student’s t-test (with Welch corrections if necessary) was used to assess the differences between the groups. The Pearson regression test was used to analyze the associations between urinary cAMP and progression parameters (i.e., eGFR, htTKV, and their temporal changes). A *p*-value below 0.05 was considered significantly different.

## 3. Results

**Figure 2:**
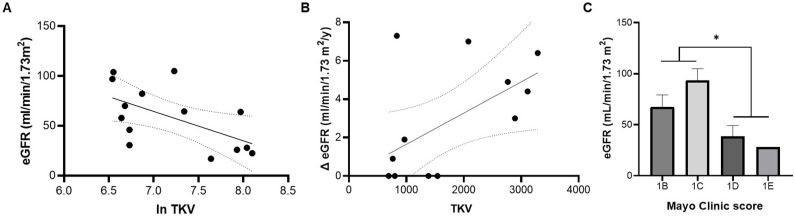
Association between mean clinical parameters of ADPKD progression. Total kidney volume (TKV) correlates with absolute (**A**) and annual change (Δ) in estimated GFR (eGFR) (**B**), and eGFR declines as the Mayo Clinic image score progresses from 1B to 1E (**C**). Regression parameters for (**A**): R = 0.58, *p* = 0.029, TKV (mL, transformed as ln) vs. eGFR; for (**B**): R = 0.57, *p* = 0.043 Δ eGFR vs. TKV. * Mayo Clinic scores 1B and 1C vs. 1D and 1E; *p* = 0.019 by unpaired *t*-test.

The mean clinical characteristics of 14 ADPKD patients aged 44 ± 2 years and predominance of female participants (10 out of 14) were moderate CKD (eGFR: 58 ± 8 mL/min/1.73 m^2^), enlarged kidneys (1.6 ± 0.3 L of TKV), and normal blood pressure (126 ± 3 and 75 ± 2 mmHg for systolic and diastolic blood pressure values, respectively), mostly (10/14 patients) due to renin–angiotensin–aldosterone system (RAAS) antagonism treatment. The main progression markers were related, and they represented the cohort’s disease evolution landscape. TKV grew exponentially as eGFR declined (Figure 2A) and showed an increase proportional to the annual decrease in GFR (Figure 2B).

CKD classification according to eGFR comprised four patients in the G1 stage, four in G2, two in G3, and four in G4. According to the image classification of Mayo Clinic, which is used to select patients for clinical trials with tolvaptan, the distribution was six patients in 1B stage, two in 1C, five in 1D, and one in 1E. The Mayo Clinic image classification score reflected the decline of filtration capacity in our cohort since eGFR worsened as the Mayo Clinic image score progressed from 1B to 1E (Figure 2C).

No subjects with proteinuria (0.30 ± 0.06 g/d) or microalbuminuria (3.55 ± 2.25 mg/mmol Cr) were found.

### 3.1. Urine EV Characterization

**Figure 3:**
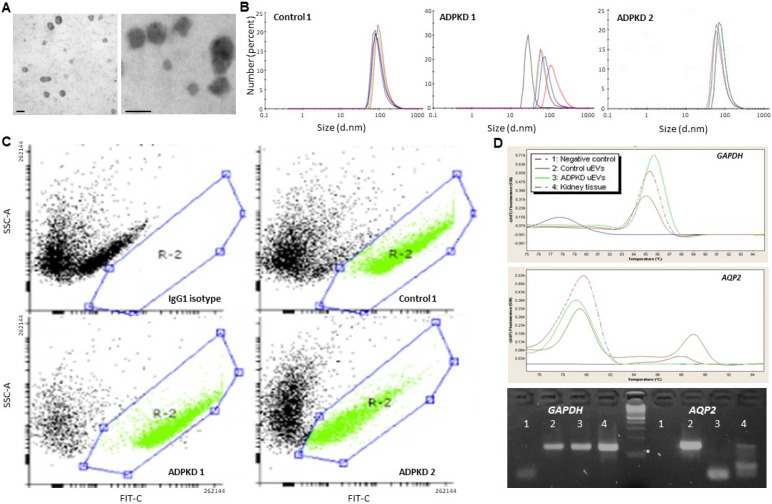
Particles obtained by an adapted centrifugation method from control and ADPKD urine samples are characterized as urinary extracellular vesicles (EVs). Morphometric and size determination by electron microscopy (**A**) and dynamic light scanning (**B**); flow cytometry for CD63 (**C**); *GAPDH* and *AQP2* mRNA detection (**D**). The lipid-bilayer spheroids ranged from 30 to 150 nm in diameter (**A**,**B**), and showed a CD63 FITC-positive signal (green) in an area (R-2) different from the IgG1 isotype in two ADPKD samples and one control sample (**C**). Real-time PCR products from control (n = 2) and ADPKD (n = 2) pooled urine EV samples showed 85.4 and 89.5 °C melting peaks for *GADPH* (**D**, upper panel) and *AQP2* (**D**, middle panel), respectively. Additionally, representative 4% agarose gel electrophoresis (**D**, lower panel) showed 137 and 146 bp bands for *GAPDH* and *AQP2* PCR products, respectively. * Denotes the 100 bp band of molecular weight marker. Scale bars: 100 nm.

The morphology and size characterization of FPCM from three control and four patient urine samples showed lipid-bilayer spheroids of 30–150 nm by EM and DLS (Figure 3A,B), whereas FC analysis showed 45 ± 5% of CD63^+^ particles in two and four control and ADPKD samples, respectively (Figure 3C). Protein and total RNA contents were 1.12 ± 0.23 μg/μL and 1.40 ± 0.29 ng/μL, respectively. To determine whether the obtained RNAs contained mRNAs, RT-qPCR for *GAPDH* and *AQP2* genes was performed in control and ADPKD pooled (n = 2 each) samples of urine. As shown in Figure 3D, PCR products with expected melting peaks and sizes on 4% agarose gels were observed for both mRNAs. According to the renal origin of the particles, *AQP2* mRNA was detected, at least in control samples. Taken together, these results show that the pellets obtained in both control and ADPKD urine specimens could be considered EVs.

### 3.2. Urine cAMP in ADPKD and Controls

**Figure 4:**
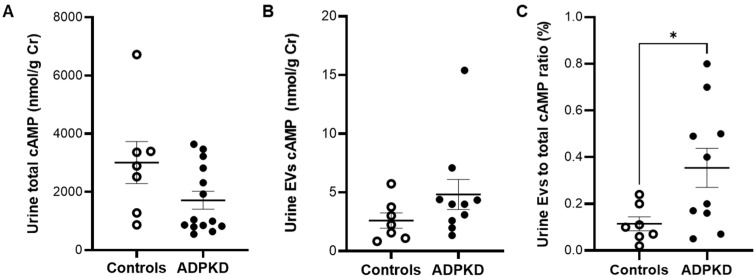
Total (**A**) and extracellular vesicles (EV) (**B**) cAMP content, and ratio of EV cAMP to total cAMP (**C**) in urine samples from controls (open circles) and ADPKD patients (black dots). * *p* = 0.02 by unpaired *t*-test with Welch’s correction.

Total cAMP urinary content was measured by the radioligand method, and no differences were found between the control (n = 7) and patient (n = 14) groups (Figure 4A). Moreover, all tested EV samples contained quantifiable levels of cAMP and no difference was observed between controls and ADPKD patients (Figure 4B). Interestingly, the proportion of cAMP contained in EVs, calculated as the ratio of EV cAMP to total cAMP ratio, showed a significantly higher content of cAMP in the EV fraction from ADPKD patients than from controls (Figure 4C).

### 3.3. Total and EV cAMP Content in ADPKD Progression

**Figure 5:**
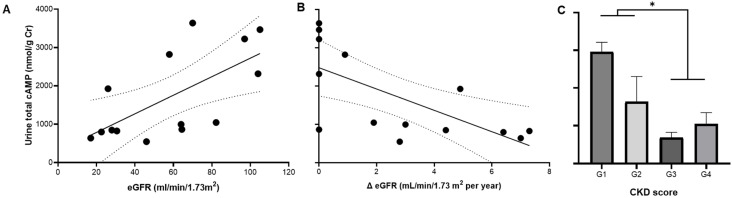
Urine total content of cAMP in ADPKD is associated with estimated GFR (eGFR) by the MDRD formula (**A**) and its annual change (Δ eGFR) (**B**), and chronic kidney disease (CKD) score (**C**). Regression parameters for (**A**): R = 0.65, *p* = 0.012; for (**B**): R = 0.67, *p* = 0.008. * CKD score G1 and G2 vs. G3 and G4; *p* = 0.021 by unpaired *t*-test with Welch’s correction.

Total urine cAMP correlated with eGFR and its annual change (*p* = 0.012 and 0.007; respectively) (Figure 5A,B). In addition, total cAMP decreased as CKD progressed (Figure 5C).

**Figure 6:**
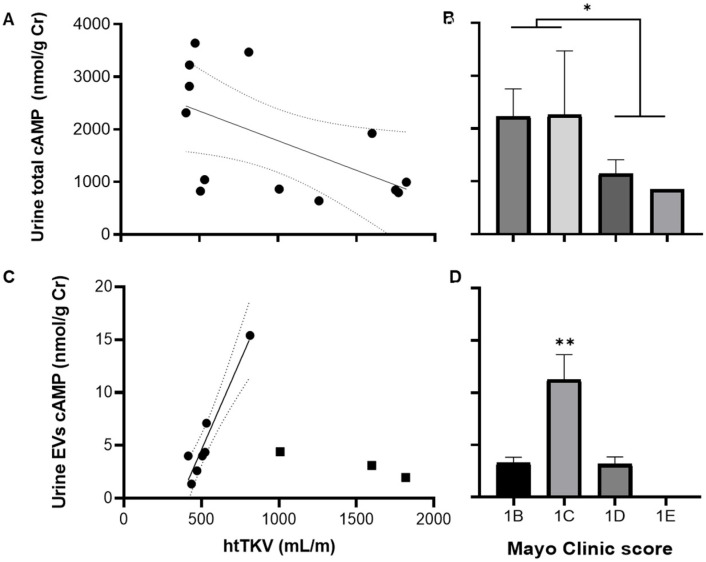
Urine total and EV cAMP contents are related to both total kidney volume (**A**,**C**) and Mayo Clinic image classification score (**B**,**D**) of ADPKD patients. Regression parameters for (**A**): R = 0.57, *p* = 0.044; for (**C**): R = 0.97, *p* = 0.0007 for black circles. * Mayo Clinic score 1B and 1C vs. 1D and 1E, *p* = 0.042 by unpaired *t*-test with Welch’s correction (**B**); ** *p* = 0.0032 by one-way ANOVA, and *p* < 0.01 vs. 1B and 1D stages by post hoc Tukey’s multiple comparisons test. htTKV: total kidney volume standardized by height.

Urine total cAMP was also related to htTKV since it decreased as the kidney grew (Figure 6A). Moreover, total cAMP also decreased as the Mayo Clinic image score progressed, and the patients at 1B and 1C (n = 8) stages showed higher levels than those in 1D and 1E (n = 6) (Figure 6B). The cAMP content in EVs showed a bimodal pattern concerning htTKV. A linear increase in cAMP content in the EVs was observed up to an approximate value of 1000 mL per meter, and then it fell abruptly at larger renal sizes (Figure 6C). This profile of cAMP content in urinary EVs was also present across the Mayo Clinic image score (Figure 6D). The urine EV to total cAMP ratio showed the same pattern across the Mayo Clinic image score as EV cAMP levels, although it did not reach significance according to the ANOVA analysis (F_(2, 8)_ = 0.998; *p* = NS). Kidney growth, expressed as a percentage of initial volume or mL/year, showed no relation to total cAMP, EV cAMP, and EV/total cAMP levels.

Blood pressure, albuminuria, and proteinuria showed no relationship with cAMP urine excretion. EV cAMP was higher in the RAAS antagonist-treated patients (angiotensin-converting enzyme inhibitors in eight and type 1 angiotensin receptor antagonists in two) than in untreated patients (4.98 ± 1.62 vs. 0.24 ± 0.13 nmol/g Cr, *p* = 0.030), but treated patients showed a more advanced stage of the disease with lower eGFR (49 ± 9 vs. 80 ± 9 mL/min/1.73 m^2^, *p* = 0.029) and higher htTKV (1100 ± 190 vs. 570 ± 150 mL/m, *p* = 0.046).

## 4. Discussion

The present results demonstrate that cAMP urine excretion correlates with the main progression markers of ADPKD and indicate that the extracellular delivery of the second messenger by EVs could reflect the architectural disturbance of the organ. In ADPKD, intracellular cAMP has been implicated in all aspects of the disease, since stimulating mural epithelial cell proliferation and transepithelial fluid secretion promotes cyst enlargement [7,25]. The seminal works of Pinto linked intracellular cAMP production with the calcium stores by adenylate cyclase and phosphodiesterase regulation in cyst growth [26,27].

Glomerular filtration rate and renal deterioration have already been associated with urine cAMP, mostly associated with concentration capacity alteration and/or vasopressin-receptor V_2_/AQP2 axis response to osmolality changes in CKD [28,29] and ADPKD [30,31]. Valuable information about cAMP and ADPKD evolution was obtained from drug testing and observational studies. TAME-PKD is a phase II clinical trial that tests metformin as a therapeutic option to ameliorate ADPKD progression, in which the association of several metabolic biomarkers and disease severity was evaluated. This study showed that urine cAMP decreases with both kidney and cystic growth and filtration function decay [32]. DIPAK consortium also found a urine cAMP-to-GFR association at baseline analysis in an observational study aimed at evaluating the role of plasma somatostatin in ADPKD progression [33]. On the other hand, an observational study in Japanese ADPKD patients did not find these associations [34]. This study had some particularities, such as a lower mean and wider range of plasma creatinine values, the use of a specific population-adapted MDRD equation to calculate eGFR, and a different range of kidney volumes that could explain the discrepancies in the results. Overall, the compelling evidence is in line with the present results that urine cAMP content paralleled the filtration decay as the disease progressed.

**Figure 7:**
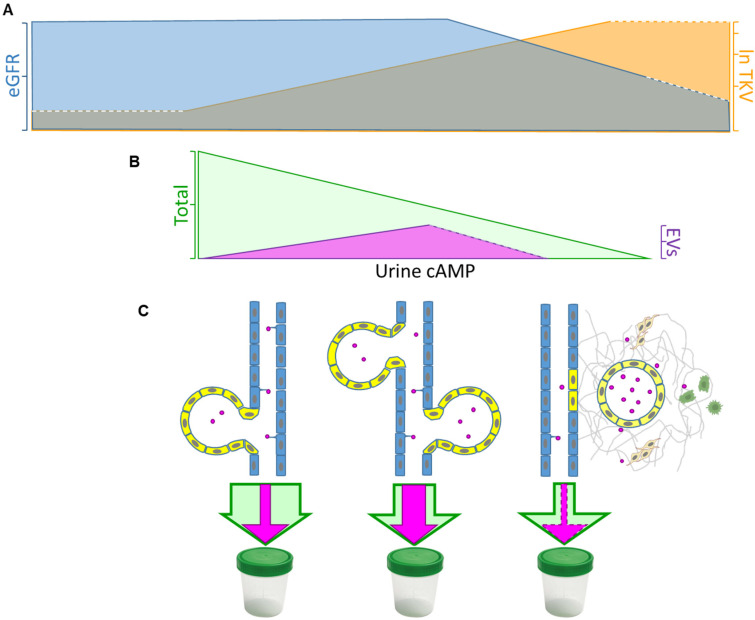
Schematic diagram illustrating the proposed mechanism of cAMP urine excretion in ADPKD progression. ADPKD progression was characterized by (**A**) a decline in eGFR (blue area) and an increase in TKV (orange area, expressed as natural logarithm), which correlated with (**B**) total urine cAMP (green area) diminution, whereas extracellular vesicle (EV) cAMP content increased at a certain point to fall at larger volumes (fuschia area). Taking into account that cystic (yellow) cells have an overproduction of cAMP, we hypothesize that (**C**) cAMP-EVs (fuschia circles) increase as the kidney grows until cysts are walled off from the tubular tree (light blue cells). According to the “cystic extracellular vesicles/exosomes theory”, EV excretion to the adjacent parenchyma gradually recruits macrophages (green cells) and activates fibroblasts (light-brown cells). Finally, the renal tissue architecture is progressively disrupted, and surrounding normal tissue is replaced by fibrosis, decreasing total (and probably EV) cAMP content in urine. Adapted from [43].

The correlation among eGFR, htTKV, and urinary cAMP may be related to the concept already described by other authors [32]: although cAMP production is expected to increase as ADPKD progresses and eGFR decays, more cysts are disconnected from functioning renal tubules and no longer communicate with the urine fluid. Then, the observation that fluid sampled directly from cysts would contain higher levels in patients with more cystic compromise and lower eGFR reflects more severe features of the disease but also indicates that urine content has diminished as fewer cysts are connected to functional renal tubules (Figure 7).

An emerging body of evidence indicates that EVs—a common term used to describe exosomes, microvesicles, and apoptotic bodies—are promising candidates for non-invasive and accessible tools for diagnosis, monitoring, and even therapeutic purposes, but some concerns have been raised about the accessibility of the multiple available methods for clinical laboratories [13]. Thus, we made some efforts to improve the quality and quantity of EV isolation in the nephrology laboratory setting by equalizing the speed-to-time ratio. In addition, we included pretreatments with DTT to disaggregate EVs and three sequential centrifugation plus filtration steps to exclude non-EV particles of the desired size. This procedure was capable of isolating vesicles that met the main criteria for EVs from ISEV and were comparable, if not even better, to particles isolated using the commercially available precipitation method [35]. The morphology and size characteristics, the presence of the transmembrane-specific CD63 protein, and the *AQP2* renal-specific mRNA content of isolated particles fulfill the current EV criteria [36]. Then, the cAMP content of these particles corresponds to EV cargo, an important discovery to expand the role of urine EVs in the cAMP signaling pathway from receptor V_2_ antagonist treatment-naïve patients and healthy subjects. It is well-recognized that microvesicles and exosomes have overlapping sizes but different origins and functions [13]. The EVs obtained in this study were 30–150 nm in diameter, and nearly half of the particles were positive for exosomal marker CD63, but the definite characterization of the cAMP-EV content origin remains an open question.

No data about cAMP in EVs from human or animal models of ADPKD were available, but an interesting study by Fonseca et al. showed that cAMP was found at higher levels in cystic than non-cystic mice [37], agreeing with the notion that the intracellular content is directly involved in cystogenesis. In addition to the intracellular cAMP pool that regulates intracellular signaling, cAMP can be extruded into the extracellular space contributing to intercellular communication [38]. In this sense, it has been proven that secreted vesicles provide extracellular cell–cell communication since cAMP-containing EVs can stimulate PKA activity in pulmonary microvascular endothelial cell cultures, establishing a novel compartment for cAMP signaling [39,40]. The present study not only demonstrates that cAMP-containing EVs are released into urine from controls and patients but also indicates that these are enriched (as suggested by the ratio of EV to total content) and correlate with kidney size in ADPKD patients. To our knowledge, no data on cAMP as cargo in EVs in disease progression have been previously reported, but it is widely accepted that intracellular cAMP is a key player in cystic expansion; therefore, our data could unravel a paracrine regulation through its transportation as EVs cargo. The compartmentalization of cAMP in urine seems to reflect a distinctive representation of kidney disease progression.

On the assumption that the urine total amount is the sum of freely excreted and EV-vehiculized content, the latter could be related to the structural burden, since it increases with kidney volume, at least up to a certain level. It is well known that cystic endowment is the driving force of kidney growth [41]; hence, EV release could be part of the underlying mechanism involved in cystic proliferation. Ding et al. elegantly showed that cystic cell-derived EVs and urinary EVs from ADPKD patients promote cyst growth in Pkd1 mutant kidneys and tridimensional cultures via the activation of PKD-associated signaling pathways in recipient cells, as well as fibroblast activation and macrophage recruitment in the neighboring tissue. This allowed the authors to proclaim the “cystic extracellular vesicles/exosomes theory”, which explains how cystic cells can affect the biology and function of neighboring cells. Additional experiments indicated that EVs secreted by cystic cells or into the urine of ADPKD patients induce the expression of miRNAs involved in cyst pathogenesis and genes associated with EV/exosome biogenesis.

Overall, evidence stresses the key role of EVs as a mediator of these processes [42]. Our results indicate a strikingly positive relationship between cAMP-EVs and kidney volumes below one liter per meter of height. as well as a drastic fall at larger volumes. Note that cystic disease progression implies that an increasing number of cysts are disconnected from the renal tubule tree. As we have already explained for urine total cAMP behavior across eGFR and htTKV, the logical consequence would be that walled-off cysts no longer excrete EVs, diminishing their urine content, whereas EVs recruit inflammation cells and fibroblasts into the renal parenchyma (Figure 7).

This proposed process seems very attractive to explain and extend the current knowledge about the role of cAMP in ADPKD evolution, but the main results need confirmation in larger studies, and several uncertainties and questions need to be further addressed. It is mandatory to determine whether the observed cAMP-EV variations imply differences in the numbers of excreted EVs or are attributable to particle cAMP concentration, and to determine how total content continues decreasing at larger volumes. Some insight into the latter is available since direct efflux and influx of cyclic nucleotide transporters have been documented in the kidney [44,45,46] and other organs [47]. Then, the non-EV cAMP concentration/content could be influenced by these transporters across the tubule system to finally contribute to the total amount of urine. Further studies based on the composition of EVs in urine and cysts and other players of extracellular mechanisms in non-EV content would be necessary to understand the complexity of cAMP regulation and its effects on ADPKD evolution.

## Data Availability

The data presented in this study are available on request from the corresponding author.
